# Associations of Agricultural Practices, Food Handling, and Socioeconomic Conditions with Household Food Security Among Urban Households in Riobamba, Ecuador

**DOI:** 10.3390/nu18142247

**Published:** 2026-07-09

**Authors:** Víctor Dante Ayaviri-Nina, Karla Carranza Mariño, Gabith Miriam Quispe Fernández, José Miguel Giner-Pérez, Ariana Saraiva, Hmidan A. Alturki, Thamer Alslamah, António Raposo

**Affiliations:** 1 Facultad de Ciencias Políticas y Administrativas, Universidad Nacional de Chimborazo (UNACH), Riobamba 060103, Ecuador; dayaviri@unach.edu.ec (V.D.A.-N.); karla.carranza@unach.edu.ec (K.C.M.); gquispe@unach.edu.ec (G.M.Q.F.); 2 Department of Applied Economics and Economic Policy, University of Alicante, 03690 Alicante, Spain; giner@ua.es; 3 Research in Veterinary Medicine (I-MVET), Faculty of Veterinary Medicine, Lisbon University Centre, Lusófona University, Campo Grande 376, 1749-024 Lisboa, Portugal; ariana.saraiva@ulusofona.pt; 4 Veterinary and Animal Research Centre (CECAV), Faculty of Veterinary Medicine, Lisbon University Centre, Lusófona University, Campo Grande 376, 1749-024 Lisboa, Portugal; 5 King Abdulaziz City for Science & Technology, Wellness and Preventive Medicine Institute—Health Sector, Riyadh 11442, Saudi Arabia; halturki@kacst.edu.sa; 6 Department of Public Health, College of Applied Medical Sciences, Qassim University, Buraydah 51452, Saudi Arabia; 4037@qu.edu.sa; 7 CBIOS (Research Center for Biosciences and Health Technologies), ECTS (School of Health Sciences and Technologies), Lusófona University, Campo Grande 376, 1749-024 Lisboa, Portugal

**Keywords:** dietary diversity, Ecuador, environmental determinants, food access, household food security, nutrition security, public health nutrition, socioeconomic determinants, sustainable food systems

## Abstract

Background: Food insecurity remains a major public health challenge in many low- and middle-income countries, where environmental and socioeconomic conditions influence household access to adequate, safe, and nutritious food. Objectives: This study aimed to evaluate the associations between environmental and socioeconomic factors and household food security in Riobamba, Ecuador. Methods: A cross-sectional survey was conducted among 382 households using the Latin American and Caribbean Food Security Scale (ELCSA), complemented by structured and Likert-scale questions addressing agricultural production practices, food handling, waste management, and food choice and dietary diversity. Household food security status was classified according to the standard ELCSA scoring procedure and subsequently grouped into categories representing adequate or inadequate household food access and consumption. Binary Logit and Probit models were estimated to identify factors associated with household food security. Results: The Probit model showed slightly better predictive performance (classification accuracy = 73.56%; Pseudo R^2^ = 0.2729; LR χ^2^ = 143.94; *p* < 0.01). Household income was the strongest predictor of food security status (Logit coefficient = 1.1977; *p* < 0.01). Among the environmental variables, agricultural production practices (β = 0.8649; *p* < 0.01), food handling practices (β = 0.3714; *p* < 0.05), and food choice and dietary diversity (β = 0.6479; *p* < 0.01) were positively associated with adequate household food access and consumption, whereas waste management practices were not significantly associated. Household size and gender were also significantly associated with food security outcomes. Conclusions: These findings indicate that household food security in urban Ecuadorian settings is associated with both environmental and socioeconomic conditions. Policies promoting sustainable agricultural practices, safe food handling, dietary diversity, and socioeconomic well-being may contribute to strengthening household food security and nutrition.

## 1. Introduction

Food insecurity remains one of the most important public health challenges worldwide, affecting billions of people and compromising access to healthy diets, nutritional adequacy, and overall well-being [1,2,3,4]. According to the Food and Agriculture Organization of the United Nations (FAO), food security exists when all people, at all times, have physical, social, and economic access to sufficient, safe, and nutritious food that meets their dietary needs and food preferences for an active and healthy life [2]. Despite global advances in food production, persistent socioeconomic inequalities, environmental pressures, and disruptions in food systems continue to pose challenges to food security in many regions of the world [1,3,5].

Food security is commonly understood through four interconnected dimensions: food availability, food access, food utilization, and food stability [2,6]. Among these dimensions, food access is particularly relevant because it is closely associated with household food consumption patterns and the ability to obtain adequate and nutritious foods. Limited access to safe foods may reduce dietary diversity and diet quality while increasing vulnerability to adverse health outcomes [7,8,9]. In recent years, the concept of nutrition security has expanded this perspective by emphasizing consistent access to safe, diverse, and nutritionally adequate diets that support health and well-being [4,10,11]. Previous studies have reported associations between food insecurity and lower dietary quality, reduced consumption of nutrient-dense foods, micronutrient inadequacies, and a greater burden of nutrition-related health conditions [8,9,12,13]. Environmental conditions have increasingly been recognized as factors associated with food security and nutrition security. Climate change, soil degradation, unsustainable agricultural practices, excessive use of agrochemicals, inadequate waste management, and poor food handling practices may be related to agricultural productivity, food quality, food safety, and the sustainability of food systems [14,15,16,17,18,19]. These environmental conditions may also be associated with the availability and accessibility of nutritious foods, particularly among households facing socioeconomic constraints [20,21,22,23]. Consequently, examining environmental conditions alongside socioeconomic characteristics may be linked to a more comprehensive understanding of household food security.

In Latin America, food insecurity remains a major public health concern despite advances in agricultural production and social protection programs. Rapid urbanization, socioeconomic inequalities, environmental degradation, and climate-related challenges continue to shape food access and consumption patterns across the region [24,25]. Previous studies have identified household income, family size, gender, and environmental conditions as factors associated with food insecurity and nutritional vulnerability [26,27,28]. These findings highlight the importance of examining the interaction between environmental and socioeconomic conditions in urban populations, where food acquisition depends largely on market access and household purchasing capacity.

The Latin American and Caribbean Food Security Scale (ELCSA) has become one of the most widely used instruments for assessing household food insecurity in the region. Its application has facilitated the identification of vulnerable populations and the examination of factors associated with food insecurity across diverse socioeconomic and geographical contexts. However, much of the available evidence has focused primarily on socioeconomic determinants, while environmental practices related to food production, food handling, waste management, and food choice and variability have received comparatively less attention, particularly in urban settings.

In Ecuador, food insecurity continues to represent an important social and public health concern, especially among economically vulnerable households. Although previous studies have documented the prevalence of food insecurity in different Ecuadorian populations, evidence regarding the association between environmental conditions and household food access and consumption remains limited, particularly in intermediate urban cities such as Riobamba [29]. Furthermore, few studies have simultaneously examined environmental and socioeconomic factors using ELCSA-based measurements and econometric approaches in urban Ecuadorian contexts.

Therefore, the present study sought to address the following research question: To what extent are environmental factors associated with household food access and consumption among households in the city of Riobamba, Ecuador?

Accordingly, the objective of this study was to evaluate the association between environmental and socioeconomic factors and household food access and consumption in the city of Riobamba, Ecuador, using the ELCSA and econometric modeling approaches. Based on previous evidence, we hypothesized that environmental conditions and socioeconomic characteristics would be significantly associated with household food security outcomes. The findings may be linked to the development of public health, nutrition, and sustainability policies aimed at improving household food security and well-being among vulnerable urban populations.

## 2. Literature Review

### 2.1. Food Security and Nutrition Security

Food security and nutrition security are interrelated concepts that extend beyond food availability and encompass access, utilization, stability, dietary quality, and nutritional adequacy. Recent research has emphasized that food security should be assessed not only in terms of caloric sufficiency but also through indicators reflecting diet quality, nutrient adequacy, sustainability, and long-term health outcomes [30,31,32]. Consequently, nutrition security has emerged as a complementary framework that recognizes the importance of ensuring consistent access to safe, diverse, and nutritionally adequate foods capable of supporting healthy and active lives [33,34,35].

Despite substantial advances in global food production over recent decades, food insecurity remains prevalent in many regions due to socioeconomic inequalities, social vulnerabilities, and structural barriers limiting access to healthy diets [30,31,32]. Food insecurity is increasingly recognized as a multidimensional phenomenon that corresponds with not only food access but also nutritional status, health outcomes, and overall well-being [34,35]. Understanding the determinants of food insecurity and nutrition security, therefore, remains a priority for public health and nutrition research.

### 2.2. Food Insecurity, Dietary Quality, and Health Outcomes

Food insecurity has been consistently associated with poorer dietary quality and reduced consumption of nutrient-dense foods. Households experiencing food insecurity often face economic constraints that limit their ability to purchase fruits, vegetables, dairy products, whole grains, and other foods essential to maintaining adequate nutritional status [36,37,38]. As a result, food-insecure populations frequently exhibit less diverse dietary patterns and lower adherence to dietary recommendations [39].

Evidence suggests that food insecurity corresponds with both the quantity and quality of food consumed. Individuals experiencing food insecurity may adopt coping strategies such as reducing meal frequency, decreasing portion sizes, or substituting nutrient-rich foods with less expensive energy-dense products [36,40]. These behaviors may be linked to inadequate nutrient intake and may strengthen the risk of nutritional deficiencies and poor health outcomes.

A growing body of literature has found significant associations between food insecurity and a wide range of adverse health outcomes. Food insecurity has been linked to obesity, cardiovascular disease, type 2 diabetes, hypertension, depression, anxiety, and poorer self-reported health status [41,42,43]. Furthermore, chronic exposure to food insecurity may negatively correspond with psychological well-being, social functioning, and overall quality of life [42].

From a public health perspective, improving food security represents an important strategy for promoting healthier dietary patterns and reducing nutrition-related health disparities. Consequently, understanding the relationship between food insecurity, dietary quality, and health outcomes is essential to the development of effective nutrition and health policies aimed at vulnerable populations [43,44].

### 2.3. Socioeconomic Determinants of Household Food Security

Food insecurity is strongly associated with socioeconomic conditions that shape households’ ability to access adequate and nutritious foods. Among these determinants, household income is consistently identified as one of the most important predictors of food security because it directly corresponds with purchasing power and the capacity to acquire diverse and healthy foods [45,46,47]. Households with limited financial resources are more likely to experience food insecurity and face difficulties in maintaining nutritionally adequate diets.

Educational attainment also plays a significant role in food security. Higher levels of education are generally associated with greater employment opportunities, higher income, support for food literacy, and healthier dietary behaviors [48,49]. Conversely, lower educational attainment may strengthen vulnerability to food insecurity by limiting access to economic resources and reducing the ability to make informed food choices.

Household composition is another important determinant of food security. Larger households often experience greater pressure on available resources, which may strengthen the likelihood of food insecurity, particularly when household income is insufficient to meet the nutritional needs of all members [50,51]. Similarly, households headed by women may face additional socioeconomic challenges related to employment opportunities, income inequality, and caregiving responsibilities, all of which can influence food access and consumption patterns [52].

Employment status and economic stability are also important factors influencing household food security. Unemployment, precarious employment conditions, and economic instability have been consistently associated with the potential to strengthen food insecurity and reduced access to healthy diets [46,53]. In contrast, stable employment and adequate income provide greater capacity to obtain nutritious foods and maintain food security over time.

Given the multifactorial nature of food insecurity, understanding the association of socioeconomic determinants is essential to designing effective interventions and public policies aimed at reducing inequalities and promoting equitable access to healthy diets among vulnerable populations [45,54].

### 2.4. Environmental Determinants of Food Security and Nutrition Security

Environmental conditions play a critical role in shaping food security and nutrition security by influencing food production, food availability, food quality, and household access to nutritious foods. Climate change, environmental degradation, biodiversity loss, soil depletion, and water scarcity have emerged as major challenges to the sustainability of food systems worldwide [55,56,57]. These environmental pressures may reduce agricultural productivity and compromise the availability of diverse and nutrient-rich foods, particularly in vulnerable populations.

Climate-related events such as droughts, floods, heatwaves, and extreme weather conditions can disrupt agricultural production systems and food supply chains, leading to reduced food availability, and may strengthen food insecurity [58,59]. In addition to corresponding with crop yields, climate variability may also be associated with the nutritional quality of foods and may strengthen the vulnerability of households dependent on local agricultural production [56,60].

Agricultural management practices are also important environmental determinants of food security. Unsustainable farming methods, excessive use of agrochemicals, inadequate soil management, and poor water resource utilization may be linked to environmental degradation and negatively correspond with long-term food production capacity [61,62]. Conversely, sustainable agricultural practices have been associated with the resilience of food systems and greater capacity to support food security under changing environmental conditions [63].

Food handling and waste management practices may further be associated with food availability and food quality throughout the food supply chain. Poor post-harvest handling, food losses, and inadequate waste management systems can reduce the efficiency of food systems and limit access to safe and nutritious foods [64,65]. Consequently, environmental management strategies aimed at improving agricultural sustainability, reducing food losses, and enhancing food system resilience are increasingly recognized as important components of food security and nutrition security policies [66].

### 2.5. Food Insecurity in Latin America and Ecuador

Food insecurity remains a significant public health challenge across Latin America despite important advances in agricultural production and economic development in several countries. The region continues to experience substantial disparities in income distribution, social protection, and access to healthy diets, which are linked to persistent food insecurity among vulnerable populations [67,68]. Recent evidence indicates that food insecurity in Latin America has been exacerbated by economic instability, climate-related events, and disruptions in food systems, disproportionately affecting low-income households and socially vulnerable groups [68,69].

The nutritional implications of food insecurity are particularly relevant in Latin America, where countries face a double burden of malnutrition characterized by the coexistence of undernutrition, micronutrient deficiencies, overweight, and obesity [70]. Limited access to nutritious foods, combined with increasing consumption of inexpensive energy-dense products, has been linked to growing health inequalities and adverse nutrition outcomes throughout the region [71].

In Ecuador, food insecurity continues to correspond with a substantial proportion of households, particularly among populations experiencing economic vulnerability and social disadvantage [72]. Previous studies have identified household income, educational attainment, employment opportunities, and access to productive resources as important determinants of food security conditions in Ecuadorian populations [73,74]. Environmental factors, including agricultural productivity, land management practices, and climate variability, may further be associated with food availability and household food access [75].

Although previous research has examined individual socioeconomic and environmental determinants of food security in Ecuador, evidence regarding the combined association of these factors on household food access and consumption in urban populations remains limited. In particular, few studies have focused on the city of Riobamba, where environmental conditions and socioeconomic inequalities may interact to influence food security outcomes. Consequently, further research is needed to better understand these relationships and support the development of effective public health, nutrition, and food security policies.

## 3. Materials and Methods

### 3.1. Study Design

A cross-sectional quantitative study was conducted among households in the urban area of Riobamba, Ecuador, to evaluate the association of environmental factors with household food access and consumption. This study was carried out in accordance with the ethical guidelines established by the National University of Chimborazo and was approved by the corresponding Research Ethics Committee on 8 February 2024 (approval code: 112-DFCPAA-UNACH). Participation was voluntary, and informed consent was obtained from all participants before data collection.

### 3.2. Data Collection and Study Population

Primary data were collected through household surveys administered in the city of Riobamba. The questionnaire included items derived from the ELCSA, developed by the Food and Agriculture Organization of the United Nations (FAO), together with structured and Likert-scale questions designed to assess environmental factors potentially associated with food access and consumption.

Secondary information was obtained from official statistical sources, scientific publications, books, and institutional reports to support the contextualization of the study.

The target population consisted of households located in the urban area of Riobamba. According to the Territorial Development and Land Use Plan (PDOT) for 2023, the city had 188,891 inhabitants. Considering an average household size of four members, the estimated number of households was 47,223.

The number of households was calculated as follows:

Population = *N* / AHS (1)

where:

- *N* = total population of Riobamba.
- AHS = average household size.

Therefore:

Population = 188,891 / 4 = 47,223 households.

The sample size was subsequently calculated using the finite population formula, resulting in a final sample of 382 households. Households were surveyed through face-to-face interviews conducted within the urban area of Riobamba. Eligible participants were adults responsible for food purchasing, preparation, or household decision making. Participation was voluntary and subject to informed consent. Although the sample size was calculated using a finite population formula, the findings should be interpreted within the context of the study population and the limitations inherent to cross-sectional household surveys.

### 3.3. Assessment of Household Food Security

Household food security was assessed using the ELCSA, a validated and widely adopted instrument developed by the Food and Agriculture Organization of the United Nations (FAO) for the assessment of household food insecurity in Latin American and Caribbean populations [76]. The ELCSA has been extensively applied in food security research and public health studies throughout the region due to its reliability and ability to identify different levels of household food insecurity [76].

The ELCSA consists of eight dichotomous questions that assess household experiences related to food access constraints resulting from economic or social limitations. Responses were coded following the standard ELCSA scoring guidelines, assigning a value of 1 to affirmative responses and 0 to negative responses, as recommended for household food insecurity assessment, assigning a value of 1 to affirmative responses and 0 to negative responses.

Total scores were obtained by summing affirmative responses and subsequently classified into four categories:

- Food security (0 affirmative responses);
- Mild food insecurity (1–3 affirmative responses);
- Moderate food insecurity (4–6 affirmative responses);
- Severe food insecurity (7–8 affirmative responses) [76].

To facilitate the econometric analysis, the original ELCSA categories were grouped into a binary outcome representing adequate versus inadequate household food access and consumption. Households classified as food-secure or mildly food-insecure were included in the adequate access and consumption category, whereas households classified as moderately or severely food-insecure were grouped into the inadequate access and consumption category. This binary specification was adopted to facilitate interpretation within the Logit and Probit modeling framework and to distinguish households with relatively adequate versus compromised food access and consumption. For econometric modeling purposes, the original ELCSA categories were grouped into a binary outcome variable. Households classified as food-secure or mildly food-insecure were coded as 1, representing the presence of food access and consumption. Households classified as moderately or severely food-insecure were coded as 0, representing the absence of adequate food access and consumption. This categorization facilitated the estimation of binary-response econometric models and has been used in previous food security studies conducted in Latin American populations.

#### Environmental Variables

Environmental variables were measured through structured questions and Likert-scale items designed to assess four dimensions: agricultural production practices, food handling practices, waste management practices, and food choice and variability. Responses were coded according to predefined scales and subsequently incorporated into the econometric analysis. Urban agricultural production refers to household practices associated with small-scale food production activities, including cultivation, food production knowledge, and related practices assessed through structured survey questions. Responses were coded using Likert-scale categories and subsequently incorporated into the econometric models.

### 3.4. Reliability Assessment

To assess the reliability of the survey instrument, Cronbach’s alpha was calculated for the overall questionnaire. The analysis yielded a Cronbach’s alpha coefficient of 0.796, indicating acceptable internal consistency, as values above 0.70 are generally considered satisfactory. The reported coefficient corresponds to the full questionnaire rather than to individual subscales.

### 3.5. Econometric Model Specification

To evaluate the association of environmental factors on household food access and consumption, two binary-response econometric models were estimated: a Logit model and a Probit model.

The dependent variable was defined as

Y = Food Access and Consumption

where:

- Y = 1, if adequate food access and consumption exist;
- Y = 0, otherwise.

The Logit model can be expressed as:

ln(P_i_/(1 − P_i_)) = β_0_ + β_1_X_1_ + β_2_X_2_ + β_3_X_3_ + β_4_X_4_ (2)

The empirical specification of the model is:

Y = f(X_1_,X_2_,X_3_,X_4_) (3)

where:

- X_1_ = agricultural production;
- X_2_ = food handling;
- X_3_ = waste management;
- X_4_ = food choice and variability.

The Probit model was estimated using the cumulative normal distribution function:

P(Y = 1∣X) = Φ(β_0_ + β_1_X_1_ + β_2_X_2_ + β_3_X_3_ + β_4_X_4_) (4)

where Φ represents the cumulative standard normal distribution function.

Both models were estimated and compared to identify the specification with the best predictive performance and classification accuracy. The final specification included environmental variables (agricultural production, food handling, waste management, and food choice and variability) and socioeconomic covariates (gender, age, marital status, monthly income, and household size).

### 3.6. Variable Definition

The dependent and independent variables included in the econometric models were selected based on the literature regarding food security, nutrition security, and environmental determinants of household food access and consumption. The dependent variable was derived from the ELCSA classification and subsequently transformed into a binary outcome variable to facilitate the estimation of the Logit and Probit models. The independent variables were designed to capture environmental dimensions potentially associated with household food security, including agricultural production practices, food handling, waste management, and food choice and variability.

Table 1 presents the coding scheme adopted for the dependent and independent variables included in the econometric analysis. Table 2 provides a detailed description of each variable, including its conceptual definition and measurement scale.

The final econometric models included four environmental variables (agricultural production practices, food handling practices, waste management practices, and food choice and variability) together with five socioeconomic covariates:

- Gender was coded as 0 = male and 1 = female.
- Age was categorized according to the survey classification.
- Marital status was entered using married/cohabiting as the reference category.
- Monthly household income was coded into the categories reported in Table 3.
- Household size was included as a categorical variable according to the number of household members.

As shown in Table 1 and Table 2, the selected variables encompass key environmental dimensions potentially associated with household food access and consumption. These variables were subsequently incorporated into the Logit and Probit models to evaluate their association with the probability of adequate household food access and consumption among households in Riobamba, Ecuador.

### 3.7. Statistical Analysis

Descriptive statistics were calculated for all study variables. Binary Logit and Probit regression models were estimated to identify environmental factors associated with household food access and consumption.

Model performance was evaluated using goodness-of-fit indicators and classification accuracy measures. The model with the highest predictive performance was selected for interpretation. Marginal effects were subsequently estimated to assess the magnitude and direction of the association of each explanatory variable on the probability of household food access and consumption.

## 4. Results

### 4.1. Descriptive Characteristics of Households

A total of 382 households participated in the study. The sociodemographic and environmental characteristics of the respondents are presented in Table 3. Most respondents were female (60.73%), while males represented 39.27% of the sample. The most frequent age group was 31–43 years (32.20%), followed by 18–30 years (30.10%). Regarding marital status, 44.76% of respondents were married. Most households reported a monthly income below USD 449.00 (39.79%), whereas 29.58% reported an income between USD 450.00 and USD 749.00. Household size was predominantly composed of two to four members (67.80%), followed by households with five to seven members (28.01%).

Regarding environmental factors, 51.31% of respondents reported limited knowledge regarding food production practices, whereas 48.69% indicated familiarity with food cultivation methods. Good food handling practices were reported by 43.72% of respondents, while 41.88% indicated adequate food waste management practices. Occasional consumption in the food choice and variability category was the most frequently reported pattern (48.69%), followed by consumption almost every day (30.37%).

### 4.2. Household Food Security Status

The distribution of households according to food security status is presented in Table 4.

Based on the ELCSA classification presented in Table 4, 53.66% of households were classified as food-secure or mildly food-insecure and were therefore considered to have adequate food access and consumption. In contrast, 46.34% of households were classified as moderately or severely food-insecure, indicating inadequate food access and consumption.

These findings suggest that nearly half of the surveyed households experience significant difficulties in obtaining sufficient and appropriate food, highlighting the persistence of food insecurity in the study population.

### 4.3. Logit and Probit Model Results

The results of the Logit and Probit estimations are presented in Table 5.

Both models were statistically significant (LR χ^2^, *p* < 0.001) and showed similar explanatory power, with pseudo R^2^ values close to 0.27. As shown in Table 5, the Probit model achieved slightly higher classification accuracy (73.56%) than the Logit model (73.30%), showing slightly higher classification performance than the Logit specification. Consequently, the Probit specification was selected for further interpretation and estimation of marginal effects.

The results revealed that gender, household income, household size, agricultural production, food handling, and food choice and variability were significantly associated with household food access and consumption. In contrast, age, marital status, and waste management were not statistically significant predictors.

### 4.4. Marginal Effects Analysis

The marginal effects derived from the Probit model are presented in Table 6.

As shown in Table 6, household income exhibited the strongest positive effect on food access and consumption, indicating that higher income levels may substantially strengthen the probability of adequate food access. Conversely, a larger household size reduced the probability of adequate food access and consumption.

Among the environmental variables, agricultural production, food handling, and food choice and variability showed significant positive effects. Greater knowledge of agricultural production practices may strengthen the probability of adequate food access and consumption by 0.1442 units. Similarly, better food handling practices may strengthen this probability by 0.0638 units, while greater food choice and variability may strengthen the probability by 0.1085 units. Although waste management presented a positive coefficient, its effect was not statistically significant.

### 4.5. Model Validation

**Figure 1:**
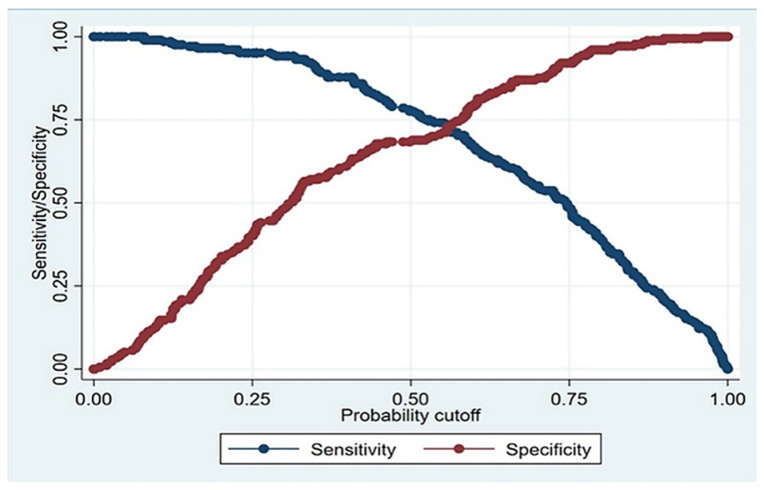
Sensitivity and specificity across probability cutoffs. Note: The graph shows the trade-off between sensitivity and specificity across different probability cutoff values used in the binary logistic regression model. The intersection point indicates the cutoff that balances both measures.

The sensitivity and specificity curves of the selected Probit model are presented in Figure 1.

As shown in Figure 1, the model achieved a sensitivity of 74.15% and a specificity of 71.19%, indicating satisfactory classification performance.

The corresponding confusion matrix is presented in Table 7. Predicted probabilities greater than or equal to 0.50 were classified as food-secure, whereas probabilities below 0.50 were classified as food-insecure.

A total of 278 out of 382 households were correctly classified according to their food access and consumption status, resulting in an overall classification accuracy of 72.77%. The model achieved a sensitivity of 74.15% and a specificity of 71.19%, indicating satisfactory predictive performance.

**Figure 2:**
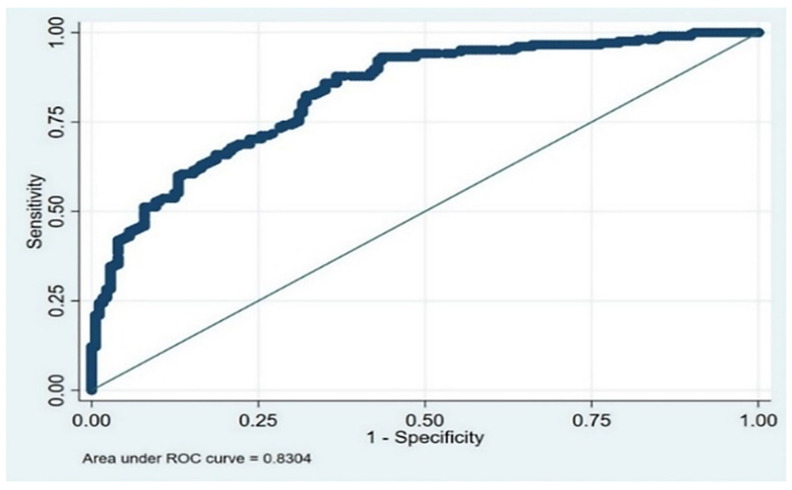
ROC curve of the logistic regression model (AUC = 0.8304). Note: The ROC curve evaluates the discriminatory performance of the logistic regression model. An AUC value of 0.8304 indicates good predictive accuracy.

The ROC diagnostic analysis is presented in Figure 2.

The area under the ROC curve (AUC) was 0.8304, indicating good discriminatory capacity and satisfactory predictive performance.

The results of the Hosmer–Lemeshow goodness-of-fit test are presented in Table 8.

The Hosmer–Lemeshow test produced a *p*-value of 0.6159, indicating no evidence of poor model fit and suggesting that the model adequately represents the observed data.

### 4.6. Predicted Probability of Household Food Access and Consumption

Using the coefficients obtained from the Probit model, the probability of adequate food access and consumption was estimated according to the cumulative normal distribution function:

P(Y = 1 | X) = Φ(β_0_ + β_1_X_1_ + β_2_X_2_ + ⋯ + β_k_X_k_) (5)

Based on the statistically significant environmental variables identified in the Probit model, the estimated equation was:

P(Y = 1 | X) = Φ(−1.0741 + 0.5097X_1_ + 0.2254X_2_ + 0.3836X_4_) (6)

where:

- X_1_ = agricultural production;
- X_2_ = food handling;
- X_4_ = food choice and variability.

Substituting the observed values used in the simulation, we obtain:

P(Y = 1 | X) = Φ[−1.0741 + 0.5097(0) + 0.2254(3) + 0.3836(2)](7)

P(Y = 1 | X) = Φ(0.3693)(8)

P(Y = 1 | X) = 0.64(9)

The estimated probability of adequate food access and consumption was 0.64, indicating a 64% likelihood that households experience adequate food access and consumption under favorable environmental conditions. This probability was derived from a hypothetical household profile characterized by positive conditions related to agricultural production, food handling, and food choice and variability. Therefore, the reported value should be interpreted as an illustrative example based on representative combinations of observed household characteristics rather than as a prediction applicable to all household profiles.

These findings suggest that favorable environmental conditions are positively associated with household food security in Riobamba. In particular, sustainable practices related to agricultural production, proper food handling, and greater diversity and choice of foods appear to contribute to support food access and consumption outcomes. The results support the study hypothesis that environmental determinants play a significant role in household food security and highlight the importance of promoting environmentally sustainable practices as a means of strengthening food security at the household level.

## 5. Discussion

### 5.1. Household Food Security and Environmental Determinants

The results indicate that agricultural production practices, food handling, and food choice and variability were positively associated with household food access and consumption in Riobamba, Ecuador. These findings are consistent with contemporary food security frameworks, which recognize that food security encompasses not only food availability but also environmental sustainability, food safety, dietary quality, and food system resilience [77,78,79,80,81]. Overall, the results suggest that environmental conditions may play an important role in shaping household food security and nutrition-related outcomes.

The positive association observed for agricultural production practices is consistent with evidence indicating that sustainable production systems may enhance food availability and strengthen the resilience of local food systems [79,80,81]. In urban settings, these practices may also reflect household engagement in food production activities that complement conventional food acquisition strategies. Households involved in agricultural production may have greater opportunities to diversify food sources, strengthen resource management capacities, and increase their engagement with local food systems. In some cases, small-scale food production may contribute to household food availability and reduce dependence on market purchases. However, given the cross-sectional nature of this study, these potential mechanisms should be interpreted as plausible explanations rather than causal pathways.

Food handling was also positively associated with food access and consumption. Previous studies have highlighted the importance of appropriate food handling practices in preserving food quality, reducing food losses, and supporting food safety throughout the food supply chain [82,83]. Improved food handling may therefore contribute to maintaining the availability and quality of foods consumed by households, thereby supporting food security outcomes.

Similarly, food choice and variability were associated with better food access and consumption outcomes. Dietary diversity is widely recognized as a key component of nutrition security and has been linked to improved nutrient adequacy and overall dietary quality [84,85]. In the present study, food choice and variability should be interpreted as indicators of dietary diversity and food selection practices rather than as direct measures of food security. Nevertheless, the observed association suggests that households with more diverse food choices may be better positioned to achieve adequate food access and consumption.

Consistent with previous research, the findings suggest that food security is influenced by multiple environmental, social, and economic factors operating simultaneously [86,87,88]. However, waste management practices were not significantly associated with food access and consumption. This lack of association may reflect measurement limitations or the fact that waste management in urban areas is largely determined by municipal infrastructure and collective service provision, thereby reducing variability at the household level. Consequently, differences in household waste management behaviors may not directly translate into observable differences in food access and consumption outcomes.

Overall, these findings support the importance of considering environmental determinants when examining household food security. The observed associations may help inform food security strategies that integrate environmental sustainability, food safety, and dietary diversity, while recognizing the complex interactions among environmental, social, and economic factors that influence household food access and consumption.

### 5.2. Socioeconomic Determinants of Food Access and Consumption

In addition to environmental variables, household income, household size, and gender were significantly associated with household food access and consumption, highlighting the multidimensional nature of food insecurity.

Household income showed the strongest positive association with food access and consumption, consistent with extensive evidence identifying income as a key determinant of food security [89,90,91]. Higher income levels may generally strengthen purchasing capacity and facilitate access to a wider variety of foods, whereas limited resources may constrain food choices and dietary quality [92,93].

Household size was negatively associated with food access and consumption. This finding agrees with previous studies showing that larger households often face greater pressure on available resources, which may reduce per capita food availability and may strengthen vulnerability to food insecurity [94,95].

Gender was also significantly associated with food access and consumption. Previous research suggests that gender-related differences in access to economic resources, employment opportunities, household decision making, and caregiving responsibilities may be associated with food security outcomes [96,97].

Together, these findings reinforce evidence that food security is closely associated with broader socioeconomic conditions, including economic resources, household composition, and social inequalities [98,99]. Therefore, interventions addressing food insecurity should consider both environmental and socioeconomic dimensions.

### 5.3. Implications for Nutrition Security and Public Health

The findings of the present study have important implications for nutrition security and public health. While food security has traditionally focused on ensuring access to sufficient quantities of food, increasing attention has been directed toward nutrition security, which emphasizes regular access to safe, nutritious, and diverse foods that promote health and well-being throughout the life course [100,101].

The positive association identified between food choice and variability and household food access and consumption reinforces the importance of dietary diversity as a fundamental component of nutrition security. Diverse diets are associated with support nutrient adequacy, better overall diet quality, and a lower risk of micronutrient deficiencies [102,103]. Consequently, interventions aimed at improving food security should move beyond food availability alone and promote access to nutritionally adequate diets capable of supporting healthy lifestyles.

The results also highlight the importance of integrating environmental sustainability into food and nutrition policies. Sustainable food systems are increasingly recognized as essential to achieving both population health and environmental objectives. Agricultural sustainability, food safety, dietary diversity, and responsible resource management are interconnected dimensions that be linked to healthier and more resilient food systems [104,105].

Food insecurity is also associated with a broad range of adverse health outcomes. Previous studies have reported significant associations between food insecurity and poor physical health, mental health disorders, psychological distress, reduced well-being, and possibly increased healthcare utilization [106,107]. Therefore, improving household food access and consumption may be linked not only to better nutritional outcomes but also to broader support of population health.

These findings are particularly relevant in Latin American contexts, where food insecurity frequently coexists with overweight, obesity, and other diet-related non-communicable diseases. This double burden of malnutrition represents one of the major public health challenges facing the region and requires integrated approaches that simultaneously address food access, dietary quality, nutrition education, and sustainable food systems [108].

Overall, the results suggest that food security interventions should incorporate nutrition-sensitive and environmentally sustainable strategies. Such approaches may be linked to healthier dietary patterns, support nutrition security, and better public health outcomes among vulnerable populations.

### 5.4. Policy Implications

The findings of this study have important implications for the development of food security, nutrition, and public health policies in Ecuador and other low- and middle-income settings facing similar environmental and socioeconomic challenges. The results demonstrate that household food access and consumption are associated with a combination of environmental and socioeconomic determinants, highlighting the need for integrated and multisectoral policy approaches.

First, interventions aimed at strengthening sustainable agricultural production should be considered a priority. Policies that support climate-resilient agricultural practices, sustainable land management, and environmentally responsible food production systems may be linked to improving food availability while simultaneously enhancing environmental sustainability [109,110]. Such strategies may also strengthen the resilience of local food systems to environmental and economic shocks.

Second, the positive association observed between food handling practices and food access highlights the importance of strengthening food safety and food loss reduction initiatives. Investments in food storage infrastructure, transportation systems, food safety education, and post-harvest management may support food quality and reduce losses throughout the food supply chain [111]. These interventions may be particularly beneficial in vulnerable populations where food losses can substantially reduce household food availability.

Third, the significant role of food choice and variability suggests that nutrition-sensitive policies should promote access to diverse and nutritionally adequate diets. Nutrition education programs, healthy food subsidies, school feeding programs, and initiatives aimed at increasing the affordability of healthy foods may be linked to improving dietary quality and nutrition security [112,113].

The results also emphasize the importance of addressing socioeconomic inequalities. Policies designed to support household income, strengthen social protection systems, and support economically vulnerable households may reduce barriers to food access and be linked to support of food security outcomes [114]. Given the strong association between income and food access identified in this study, economic interventions may have substantial benefits for household nutrition and well-being.

In addition, integrated multisectoral actions involving local governments, public health agencies, agricultural extension services, educational institutions, and community organizations may further strengthen household food security. Coordinated interventions could include expanding urban agriculture initiatives, promoting food safety and food handling education, encouraging dietary diversity, and implementing targeted programs to improve food access among vulnerable households. Such collaborative efforts may contribute to addressing the multiple environmental, social, and economic factors that influence household food access and consumption.

Finally, food security policies should be integrated within broader public health and sustainable development strategies. The achievement of food security, nutrition security, environmental sustainability, and health equity requires coordinated actions across agriculture, health, education, social protection, and environmental sectors [115]. Such integrated approaches may be linked to the development of more resilient food systems and healthier populations.

Overall, the findings support the implementation of comprehensive policies that simultaneously address environmental sustainability, socioeconomic vulnerability, and nutrition security in order to support household food access and consumption.

### 5.5. Strengths and Limitations

This study presents several strengths that be linked to the understanding of food security determinants in urban Ecuadorian households. First, the study simultaneously examined environmental and socioeconomic factors associated with household food access and consumption, providing a more comprehensive perspective on food security than approaches focused exclusively on economic determinants. Second, the use of the ELCSA, a widely used and validated instrument for assessing food security in the region, enhances the comparability of the findings with previous studies conducted in Latin America. Third, the application of both Logit and Probit econometric models allowed for the evaluation of the robustness and consistency of the estimated associations. Finally, the inclusion of environmental variables related to agricultural production, food handling, waste management, and food choice and variability can be linked to the growing literature linking environmental sustainability, food systems, and nutrition security.

Despite these strengths, several limitations should be acknowledged. The cross-sectional design of this study does not allow causal relationships to be established between the explanatory variables and food access and consumption. Consequently, the observed associations should be interpreted as correlational rather than causal. In addition, this study relied on self-reported information, which may be subject to recall bias and reporting bias. Furthermore, detailed information regarding item-level missing responses and non-response patterns was not available in the original dataset. Consequently, the potential influence of missing data on the reported associations could not be formally evaluated. Another limitation is that the present research study was conducted exclusively in the city of Riobamba; therefore, the findings may not be fully generalizable to other regions of Ecuador with different socioeconomic, cultural, and environmental characteristics. The binary classification of food security simplified the interpretation of household food access outcomes and facilitated estimation using Logit and Probit models. However, this approach may have reduced information regarding differences between moderate and severe food insecurity. Consequently, the findings should be interpreted as reflecting broader patterns of household food access rather than distinctions across all levels of food insecurity. Although classification accuracy exceeded 73%, a proportion of households remained incorrectly classified. Consequently, the models should be interpreted primarily as explanatory tools for examining associations rather than as instruments for individual-level targeting or prediction. In addition, the original analytical database did not permit recalculation of reliability indicators such as Cronbach’s alpha or the implementation of additional psychometric analyses for individual questionnaire dimensions. Therefore, the measurement properties of specific constructs could not be reassessed within the scope of the present study.

Furthermore, although this study incorporated several relevant environmental and socioeconomic determinants, other factors potentially associated with household food security—such as food prices, market accessibility, educational attainment, dietary behaviors, social support networks, household nutritional knowledge, employment conditions, and access to credit—were not available in the original dataset and therefore could not be included in the econometric models. Similarly, the probability simulations presented in this study were intended for illustrative purposes and were based on representative combinations of observed household characteristics. Consequently, they should not be interpreted as predictions applicable to all household profiles. Future studies should explore these dimensions and consider longitudinal approaches to better understand the dynamic relationships between environmental conditions, socioeconomic factors, and food security outcomes. Additional model comparison statistics such as AIC and BIC could not be recalculated because the original analytical database was unavailable.

Nevertheless, this study provides valuable evidence regarding environmental and socioeconomic factors associated with household food access and consumption in an urban Ecuadorian context and offers useful insights for the development of food security, nutrition, and public health policies. Future longitudinal and prospective studies are needed to better understand the temporal relationships between environmental conditions, socioeconomic characteristics, and household food security outcomes. Additional research incorporating broader socioeconomic variables, psychometric validation procedures, and alternative modeling approaches may further strengthen our understanding of household food security dynamics in Ecuadorian urban settings. The urban focus of this study should also be considered when interpreting the findings. Household food access dynamics in Riobamba may differ from those observed in rural or other urban contexts with distinct socioeconomic, cultural, and environmental conditions. Consequently, the generalizability of the results beyond the study area may be limited.

Additionally, the reliance on self-reported information may have introduced recall bias, reporting bias, and social desirability bias. These factors may affect the accuracy of responses related to environmental practices and household food access.

Finally, the possibility of omitted-variable bias cannot be excluded. Variables such as educational attainment, market accessibility, employment conditions, access to credit, food prices, and nutrition knowledge were not available in the original dataset and therefore could not be incorporated into the econometric models. As a result, some observed associations may partially reflect the influence of unmeasured factors.

## 6. Conclusions

This study examined the association of environmental and socioeconomic factors on household food access and consumption in the city of Riobamba, Ecuador. The findings suggest that both environmental and socioeconomic factors are significantly associated with household food security outcomes.

Among the environmental factors, agricultural production practices, food handling, and food choice and variability were positively associated with household food access and consumption, highlighting the importance of sustainable food system components in supporting food security. In addition, socioeconomic factors, particularly household income, household size, and gender, were significantly associated with food access and consumption, emphasizing the multidimensional nature of food insecurity.

The results reinforce the growing recognition that food security extends beyond food availability and should be considered within broader frameworks that incorporate nutrition security, dietary quality, environmental sustainability, and social equity. Households experiencing favorable environmental conditions and supportive socioeconomic circumstances were more likely to achieve adequate food access and consumption, suggesting that integrated interventions are required to address the multiple determinants of food insecurity.

From a public health perspective, the findings underscore the importance of promoting sustainable agricultural practices, improving food handling and food safety conditions, supporting dietary diversity, and reducing socioeconomic inequalities. Such actions may be linked not only to support of food security but also to better nutrition outcomes, healthier dietary patterns, and enhanced population well-being.

Overall, this study provides evidence that environmental sustainability and socioeconomic development are closely interconnected with household food security. The findings support the implementation of multisectoral policies that simultaneously address environmental, nutritional, economic, and social determinants to strengthen food security, nutrition security, and public health outcomes in Ecuador and similar settings.

Future research should explore these relationships using longitudinal designs and broader geographic coverage to further understand the complex interactions between environmental conditions, socioeconomic factors, and food security outcomes. A key contribution of this study is the simultaneous examination of environmental and socioeconomic factors associated with household food security in an urban Ecuadorian context using ELCSA-based measures and econometric modeling.

## Figures and Tables

**Table 1:** Variable coding.

Variable	Parameter	Type	Coding
Food access and consumption	Y	Dependent	Access and Consumption = 1;
			No Access and Consumption = 0
Agricultural production	X_1_	Independent	Yes = 1; No = 0
Food handling	X_2_	Independent	Very Good = 4; Good = 3; Neither Good Nor Bad = 2;
			Bad = 1; Very Bad = 0
Waste management	X_3_	Independent	Very Suitable = 4; Suitable = 3; Neutral = 2; Inadequate = 1;
			Very Inadequate = 0
Food choice and variability	X_4_	Independent	Every day = 4; Almost Every Day = 3; Occasionally = 2;
			Almost Never = 1; Never = 0

Note: Own elaboration based on data collected during the study in 2024.

**Table 2:** Description of variables.

Variable	Type	Description
Food access and consumption	Dependent	Household food security status measured using ELCSA and categorized into binary outcomes
Agricultural production	Independent	Agricultural practices related to fertilizer use, soil recovery, and crop management
Food handling	Independent	Food handling practices including storage, transportation, and commercialization
Waste management	Independent	Household waste management and classification practices
Food choice and variability	Independent	Diversity and frequency of food consumption patterns

Note: Own elaboration, 2024.

**Table 3:** Descriptive characteristics of respondents and households.

Variable	Category	*n*	%
Gender	Female	232	60.73
	Male	150	39.27
Age	18–30 years	115	30.10
	31–43 years	123	32.20
	44–56 years	102	26.70
	57–69 years	40	10.47
	≥70 years	2	0.52
Marital status	Married	171	44.76
	Single	148	38.74
	Divorced	58	15.18
	Widowed	5	1.31
Monthly income	<USD 449	152	39.79
	USD 450–749	113	29.58
	USD 750–1049	71	18.59
	USD 1050–1349	25	6.54
	≥USD 1350	21	5.50
Household size	2–4 members	259	67.80
	5–7 members	107	28.01
	8–10 members	15	3.93
	≥11 members	1	0.26
Agricultural production knowledge	Yes	186	48.69
	No	196	51.31
Food handling knowledge	Very good	50	13.09
	Good	167	43.72
	Neutral	142	31.17
	Poor	21	5.50
	Very poor	2	0.52
Waste management	Very suitable	21	5.50
	Suitable	160	41.88
	Neutral	119	31.15
	Inadequate	82	21.47
	Very inadequate	0	0.00
Food choice and variability	Every day	41	10.73
	Almost every day	116	30.37
	Occasionally	186	48.69
	Almost never	37	9.69
	Never	2	0.52

Note: Values are presented as frequency (*n*) and percentage (%). Total sample size = 382 households.

**Table 4:** Household food security status according to ELCSA classification.

Food Security Category	Frequency (%)
Food security + mild food insecurity	53.66
Moderate + severe food insecurity	46.34

Note: Household food security status was classified according to the ELCSA scale and grouped into two categories for analysis. Own elaboration based on study data collected in 2024.

**Table 5:** Logit and Probit model estimation.

Variables	Logit	Probit
Gender	−1.0869 ***	−0.6321 ***
Age	0.1312	0.0835
Marital status	0.2480	0.1488
Income	1.1977 ***	0.7053 ***
Household members	−0.9435 ***	−0.5737 ***
Agricultural production	0.8649 ***	0.5097 ***
Food handling	0.3714 **	0.2254 **
Waste management	0.2353	0.1380
Food choice and variability	0.6479 ***	0.3836 ***
Constant	−1.6560	−1.0741
Pseudo R^2^	0.2721	0.2729
LR Chi^2^	143.51 ***	143.94 ***
Classification accuracy	73.30%	73.56%

Note: Dependent variable: household food access and consumption (1 = access and consumption; 0 = no access and consumption). Coefficients correspond to Logit and Probit models. *** *p* < 0.01; ** *p* < 0.05. Own elaboration based on study data collected in 2024.

**Table 6:** Marginal effects of the Probit model.

Variables	Marginal Effect
Gender	−0.1789 ***
Age	0.0236
Marital status	0.0421
Income	0.1996 ***
Household members	−0.1623 ***
Agricultural production	0.1442 ***
Food handling	0.0638 **
Waste management	0.0390
Food choice and variability	0.1085 ***
C	−1.0741
Pseudo R^2^	0.2729
LR Chi^2^	143.94 ***

Note: Marginal effects were calculated from the Probit model and represent the change in the probability of household food access and consumption associated with a one-unit increase in each explanatory variable, holding the other variables constant. *** *p* < 0.01; ** *p* < 0.05. Own elaboration based on study data collected in 2024.

**Table 7:** Confusion matrix and classification performance of the Probit model.

**Observed Classification**	**Predicted Access and Consumption**	**Predicted No Access and Consumption**	**Total**
Access and Consumption	152	53	205
No Access and Consumption	51	126	177
Total	203	179	382
**Model Performance Indicators**			
**Indicator**		**Value (%)**	
Sensitivity		74.15	
Specificity		71.19	
Correct Classification Rate		72.77	

Note: The confusion matrix compares observed and predicted classifications generated by the Probit model. Sensitivity corresponds to the true-positive rate, specificity to the true-negative rate, and the correct classification rate to the percentage of observations correctly classified. Own elaboration based on study data collected in 2024.

**Table 8:** Hosmer–Lemeshow goodness-of-fit test.

Statistic	Value
Number of observations	382
Number of groups	10
Hosmer–Lemeshow χ^2^	6.28
Prob > χ^2^	0.6159

Note: The Hosmer–Lemeshow χ^2^ statistic assesses the calibration of the Probit model by comparing observed and expected frequencies across groups of predicted probabilities. The non-significant result (*p* = 0.6159) suggests no evidence of lack of fit. Own elaboration based on study data collected in 2024.

## Data Availability

The original contributions presented in this study are included in the article. Further inquiries can be directed to the corresponding author.

## References

[1] Clapp J., Moseley W.G., Burlingame B., Termine P. (2022). The Case for a Six-Dimensional Food Security Framework. Food Policy.

[2] FAO, IFAD, UNICEF, WFP, WHO (2024). The State of Food Security and Nutrition in the World 2024: Financing to End Hunger, Food Insecurity and Malnutrition in All Its Forms.

[3] Smith M.D., Rabbitt M.P., Coleman-Jensen A. (2017). Who Are the World’s Food Insecure? New Evidence from the Food Insecurity Experience Scale. World Dev..

[4] Mozaffarian D., Fleischhacker S., Andrés J.R. (2021). Prioritizing Nutrition Security in the United States. JAMA.

[5] Abay K.A., Breisinger C., Glauber J., Kurdi S., Laborde D., Siddig K. (2023). The Russia–Ukraine War: Implications for Global and Regional Food Security and Potential Policy Responses. Glob. Food Sec..

[6] Iversen T.O., Westengen O.T., Jerven M. (2023). The History of Hunger: Counting Calories to Make Global Food Security Legible. World Dev. Perspect..

[7] Hanson K.L., Connor L.M. (2014). Food Insecurity and Dietary Quality in US Adults and Children: A Systematic Review. Am. J. Clin. Nutr..

[8] Leung C.W., Tester J.M. (2019). The Association between Food Insecurity and Diet Quality. J. Acad. Nutr. Diet..

[9] Gundersen C., Ziliak J.P. (2015). Food Insecurity and Health Outcomes. Health Aff..

[10] Urasaki Y., Le T.T. (2022). A composition of phytonutrients for glycemic and weight management. Nutrients.

[11] Pérez-Escamilla R. (2024). Food and nutrition security definitions, constructs, frameworks, measurements, and applications: Global lessons. Front. Public Health.

[12] Pourmotabbed A., Moradi S., Babaei A., Ghavami A., Mohammadi H., Jalili C., E Symonds M., Miraghajani M. (2020). Food Insecurity and Mental Health: A Systematic Review and Meta-Analysis. Public Health Nutr..

[13] Martinez S.M., Frongillo E.A., Leung C., Ritchie L. (2020). No food for thought: Food insecurity is related to poor mental health and lower academic performance among students in California’s public university system. J. Health Psychol..

[14] Chandio A.A., Jiang Y., Amin A., Ahmad M., Akram W., Ahmad F. (2023). Climate Change and Food Security of South Asia: Fresh Evidence from a Policy Perspective Using Novel Empirical Analysis. J. Environ. Plan. Manag..

[15] Singh B.K., Delgado-Baquerizo M., Egidi E., Guirado E., Leach J.E., Liu H., Trivedi P. (2023). Climate Change Be related toon Plant Pathogens, Food Security and Paths Forward. Nat. Rev. Microbiol..

[16] Mirón I.J., Linares C., Díaz J. (2023). The Association of Climate Change on Food Production and Food Safety. Environ. Res..

[17] El Bilali H., Bassole I.H.N., Dambo L., Berjan S. (2020). Climate Change and Food Security. Agric. For..

[18] Adesete A.A., Olanubi O.E., Dauda R.O. (2023). Climate Change and Food Security in Selected Sub-Saharan African Countries. Environ. Dev. Sustain..

[19] Dasgupta S., Robinson E.J.Z. (2022). Attributing Changes in Food Insecurity to a Changing Climate. Sci. Rep..

[20] Hasegawa T., Sakurai G., Fujimori S., Takahashi K., Hijioka Y., Masui T. (2021). Extreme Climate Events May strengthen Risk of Global Food Insecurity and Adaptation Needs. Nat. Food.

[21] Hadley K., Wheat S., Rogers H.H., Balakumar A., Gonzales-Pacheco D., Davis S.S., Sorensen C. (2023). Mechanisms Underlying Food Insecurity in the Aftermath of Climate-Related Shocks: A Systematic Review. Lancet Planet. Health.

[22] Schattman R.E., Rowland D.L., Kelemen S.C. (2023). Sustainable and Regenerative Agriculture: Tools to Address Food Insecurity and Climate Change. J. Soil Water Conserv..

[23] Guell C., Saint Ville A., Anderson S.G., Murphy M.M., Iese V., Kiran S., Unwin N. (2024). Small Island Developing States: Addressing the Intersecting Challenges of Non-Communicable Diseases, Food Insecurity and Climate Change. Lancet Diabetes Endocrinol..

[24] Pérez-Escamilla R., Gubert M.B., Rogers B., Hromi-Fiedler A. (2017). Food Security Measurement and Governance: Assessment of the Usefulness of Diverse Food Insecurity Indicators for Policy Makers. Glob. Food Sec..

[25] UNICEF, WFP, WHO, FAO, IFAD (2023). Regional Overview of Food Security and Nutrition in Latin America and the Caribbean 2023: Statistics and Trends.

[26] Kolog J.D., Asem F.E., Mensah-Bonsu A. (2023). The State of Food Security and Its Determinants in Ghana: An Ordered Probit Analysis. Sci. Afr..

[27] Moon M.P. (2024). How Does Climate Change Correspond with the Food Security and Vulnerability of Women? A Systematic Review of Gender Perspectives. Front. Clim..

[28] Perez-Escamilla R., Bermudez O., Buccini G.S., Kumanyika S., Lutter C.K., Monsivais P., Victora C. (2018). Nutrition Disparities and the Global Burden of Malnutrition. BMJ.

[29] Andrade C., Ayaviri V. (2017). Cuestiones Ambientales y Seguridad Alimentaria en el Cantón Guano, Ecuador. Inf. Tecnol..

[30] Jones A.D., Ngure F.M., Pelto G., Young S.L. (2013). What Are We Assessing When We Measure Food Security? A Compendium and Review of Current Metrics. Adv. Nutr..

[31] Fanzo J., Haddad L., McLaren R., Marshall Q., Davis C., Herforth A., Jones A., Beal T., Tschirley D., Bellows A. (2020). The Food Systems Dashboard Is a New Tool to Inform Better Food Policy. Nat. Food.

[32] Béné C., Fanzo J., Prager S.D., Achicanoy H.A.E., Mapes B.R., Alvarez Toro P., Bonilla Cedrez C. (2020). Global Drivers of Food System (Un)Sustainability: A Multi-Country Correlation Analysis. PLoS ONE.

[33] Anderson S.A. (1990). Core Indicators of Nutritional State for Difficult-to-Sample Populations. J. Nutr..

[34] Seligman H.K., Schillinger D. (2010). Hunger and Socioeconomic Disparities in Chronic Disease. N. Engl. J. Med..

[35] Cook J.T., Frank D.A. (2008). Food Security, Poverty, and Human Development in the United States. Ann. N. Y. Acad. Sci..

[36] Leung C.W., Epel E.S., Ritchie L.D., Crawford P.B., Laraia B.A. (2014). Food Insecurity Is Inversely Associated with Diet Quality of Lower-Income Adults. J. Acad. Nutr. Diet..

[37] Darmon N., Drewnowski A. (2008). Does Social Class Predict Diet Quality?. Am. J. Clin. Nutr..

[38] Rao M., Afshin A., Singh G., Mozaffarian D. (2013). Do Healthier Foods and Diet Patterns Cost More Than Less Healthy Options?. BMJ Open.

[39] Drewnowski A., Specter S.E. (2004). Poverty and Obesity: The Role of Energy Density and Energy Costs. Am. J. Clin. Nutr..

[40] Althoff R.R., Ametti M., Bertmann F. (2016). The Role of Food Insecurity in Developmental Psychopathology. Prev. Med..

[41] Gregory C.A., Coleman-Jensen A. Food Insecurity, Chronic Disease, and Health Among Working-Age Adults. USDA Economic Research Report 2017, No. 235. https://ers.usda.gov/sites/default/files/_laserfiche/publications/84467/ERR-235_Summary.pdf?v=27015.

[42] Weaver L.J., Hadley C. (2009). Moving Beyond Hunger and Nutrition: A Systematic Review of the Evidence Linking Food Insecurity and Mental Health in Developing Countries. Ecol. Food Nutr..

[43] Gundersen C., Kreider B., Pepper J. (2011). The Economics of Food Insecurity in the United States. Appl. Econ. Perspect. Policy.

[44] Loopstra R. (2018). Interventions to Address Household Food Insecurity in High-Income Countries. Proc. Nutr. Soc..

[45] Nord M., Andrews M., Carlson S. (2009). Household Food Security in the United States, 2008.

[46] Coleman-Jensen A., Rabbitt M.P., Gregory C.A., Singh A. (2023). Household Food Security in the United States in 2022.

[47] Tarasuk V., Mitchell A., Dachner N. (2016). Household Food Insecurity in Canada, 2014. Research to Identify Policy Options to Reduce Food Insecurity (PROOF).

[48] Smith L.C., El Obeid A.E., Jensen H.H. (2000). The Geography and Causes of Food Insecurity in Developing Countries. Agric. Econ..

[49] Mutisya M., Ngware M.W., Kabiru C.W., Kandala N.B. (2016). The Effect of Education on Household Food Security in Two Informal Urban Settlements in Kenya. BMC Public Health.

[50] Bashir M.K., Schilizzi S., Pandit R. (2012). The Determinants of Rural Household Food Security in the Punjab, Pakistan.

[51] Sekhampu T.J. (2013). Determination of the Factors Correspond withing the Food Security Status of Households in Bophelong, South Africa. Int. Bus. Econ. Res. J..

[52] Mallick D., Rafi M. (2010). Are Female-Headed Households More Food Insecure? Evidence from Bangladesh. World Dev..

[53] Huang J., Kim Y., Birkenmaier J., Kim M. (2016). Unemployment and Household Food Hardship in the Economic Recession. Public Health Nutr..

[54] Pinstrup-Andersen P. (2009). Food Security: Definition and Measurement. Food Secur..

[55] Wheeler T., von Braun J. (2013). Climate Change Be related to on Global Food Security. Science.

[56] Myers S.S., Smith M.R., Guth S., Golden C.D., Vaitla B., Mueller N.D., Dangour A.D., Huybers P. (2017). Climate Change and Global Food Systems: Potential Be related to on Food Security and Undernutrition. Annu. Rev. Public Health.

[57] Fanzo J., Davis C., McLaren R., Choufani J. (2018). The Effect of Climate Change across Food Systems: Implications for Nutrition Outcomes. Glob. Food Sec..

[58] Morton J.F. (2007). The Be related to of Climate Change on Smallholder and Subsistence Agriculture. Proc. Natl. Acad. Sci. USA.

[59] Lobell D.B., Schlenker W., Costa-Roberts J. (2011). Climate Trends and Global Crop Production since 1980. Science.

[60] Beach R.H., Sulser T.B., Crimmins A., Cenacchi N., Cole J., Fukagawa N.K., Mason-D’Croz D., Myers S.S., Sarofim M.C., Smith M. (2019). Combining the Effects of May strengthend Atmospheric Carbon Dioxide on Protein, Iron, and Zinc Availability and Projected Climate Change on Global Diets. Lancet Planet. Health.

[61] Tilman D., Balzer C., Hill J., Befort B.L. (2011). Global Food Demand and the Sustainable Intensification of Agriculture. Proc. Natl. Acad. Sci. USA.

[62] Foley J.A., Ramankutty N., Brauman K.A., Cassidy E.S., Gerber J.S., Johnston M., Mueller N.D., O’Connell C., Ray D.K., West P.C. (2011). Solutions for a Cultivated Planet. Nature.

[63] Pretty J., Bharucha Z.P. (2014). Sustainable Intensification in Agricultural Systems. Ann. Bot..

[64] Gustavsson J., Cederberg C., Sonesson U. (2011). Global Food Losses and Food Waste.

[65] FAO (2019). The State of Food and Agriculture 2019: Moving Forward on Food Loss and Waste Reduction.

[66] Springmann M., Clark M., Mason-D’Croz D., Wiebe K., Bodirsky B.L., Lassaletta L., de Vries W., Vermeulen S.J., Herrero M., Carlson K.M. (2018). Options for Keeping the Food System within Environmental Limits. Nature.

[67] Pérez-Escamilla R., Vilar-Compte M., Rhodes E., Sarmiento O.L. (2017). Food Insecurity Measurement and Governance in Latin America and the Caribbean. Rev. Panam. Salud Publica.

[68] Food and Agriculture Organization of the United Nations (FAO) (2024). Panorama Regional de La Seguridad Alimentaria Y La Nutrición En América Latina Y El Caribe 2024.

[69] Economic Commission for Latin America and the Caribbean (ECLAC), Food and Agriculture Organization of the United Nations (FAO) (2022). Food Security Under the New Development Model in Latin America and the Caribbean.

[70] Rivera J.A., Pedraza L.S., Martorell R., Gil A. (2014). Introduction to the Double Burden of Undernutrition and Excess Weight in Latin America. Am. J. Clin. Nutr..

[71] Popkin B.M., Reardon T. (2018). Obesity and the Food System Transformation in Latin America. Obes. Rev..

[72] Freire W.B., Silva-Jaramillo K.M., Ramírez-Luzuriaga M.J., Belmont P., Waters W.F. (2014). The Double Burden of Malnutrition in Ecuador. Am. J. Clin. Nutr..

[73] Cordero-Ahiman O.V., Vanegas J.L., Franco-Crespo C., Beltrán-Romero P., Quinde-Lituma M.E. (2021). Factors That Be associated with Food Insecurity in Rural Households in Ecuador. Sustainability.

[74] Ayaviri-Nina V.D., Quispe-Fernández G.M., Vanegas J.L., Ortega-Mejía V., Cordero-Ahiman O.V. (2022). Importance of Purchasing Power and Education in the Food Security of Families in Rural Areas—Case Study: Chambo, Ecuador. Sustainability.

[75] Rampazzo G., Zironi E., Pagliuca G., Gazzotti T. (2022). Analysis of Cobalamin (Vit B12) in Ripened Cheese by Ultra-High-Performance Liquid Chromatography Coupled with Mass Spectrometry. Foods.

[76] ELCSA (2012). Escala Latinoamericana Y Caribeña de Seguridad Alimentaria (ELCSA): Manual de Uso Y Aplicaciones.

[77] High Level Panel of Experts on Food Security and Nutrition (HLPE) (2020). Food Security and Nutrition: Building a Global Narrative Towards 2030.

[78] Webb P., Benton T.G., Beddington J., Flynn D., Kelly N.M., Thomas S.M. (2020). The Urgency of Food System Transformation Is Now Irrefutable. Nat. Food.

[79] Willett W., Rockström J., Loken B., Springmann M., Lang T., Vermeulen S., Garnett T., Tilman D., DeClerck F., Wood A. (2019). Food in the Anthropocene: The EAT–Lancet Commission on Healthy Diets from Sustainable Food Systems. Lancet.

[80] Swinburn B.A., Kraak V.I., Allender S., Atkins V.J., Baker P.I., Bogard J.R., Brinsden H., Calvillo A., De Schutter O., Devarajan R. (2019). The Global Syndemic of Obesity, Undernutrition, and Climate Change. Lancet.

[81] Berry E.M., Dernini S., Burlingame B., Meybeck A., Conforti P. (2015). Food Security and Sustainability: Can One Exist without the Other?. Public Health Nutr..

[82] Nordhagen S., Hoffmann V., Brückner G.K. (2022). Food Safety and Nutrition: Protecting Consumers While Improving Diets. Ann. N. Y. Acad. Sci..

[83] Grace D. (2015). Food Safety in Low- and Middle-Income Countries. Int. J. Environ. Res. Public Health.

[84] Miller V., Webb P., Micha R., Mozaffarian D. (2020). Defining Diet Quality: A Synthesis of Dietary Quality Metrics and Their Validity. Nutrients.

[85] Herforth A., Bai Y., Venkat A., Mahrt K., Ebel A., Masters W.A. (2020). Cost and Affordability of Healthy Diets Across and Within Countries.

[86] Jones A.D. (2017). Food Insecurity and Mental Health Status: A Global Analysis of 149 Countries. Am. J. Prev. Med..

[87] Barrett C.B. (2022). Actions now can curb food systems fallout from COVID-19. Nat. Food.

[88] Fan S., Yosef S., Pandya-Lorch R. (2019). Agriculture for Support Nutrition: Seizing the Momentum.

[89] Coleman-Jensen A., Gregory C., Singh A. (2024). Household Food Security in the United States in 2023.

[90] Mitu M.M.P., Islam K., Sarwar S., Ali M., Amin M.R. (2022). Spatial Differences in Diet Quality and Economic Vulnerability to Food Insecurity in Bangladesh: Results from the 2016 Household Income and Expenditure Survey. Sustainability.

[91] Frelat R., Lopez-Ridaura S., Giller K.E., Herrero M., Douxchamps S., Andersson Djurfeldt A., Erenstein O., Henderson B., Kassie M., Paul B.K. (2016). Drivers of Household Food Availability in Sub-Saharan Africa Based on Big Data from Small Farms. Proc. Natl. Acad. Sci. USA.

[92] Darmon N., Drewnowski A. (2015). Contribution of Food Prices and Diet Cost to Socioeconomic Disparities in Diet Quality and Health. Nutr. Rev..

[93] Drewnowski A. (2010). The Cost of US Foods as Related to Their Nutritive Value. Am. J. Clin. Nutr..

[94] Bashir M.K., Schilizzi S., Pandit R. (2012). The Determinants of Rural Household Food Security in the Punjab, Pakistan: An Econometric Analysis.

[95] Aidoo R., Mensah J.O., Tuffour T. Determinants of Household Food Security in the Sekyere-Afram Plains District of Ghana. Proceedings of the 1st Annual International Interdisciplinary Conference (AIIC 2013).

[96] Quisumbing A.R., Brown L.R., Feldstein H.S., Haddad L., Peña C. (1995). Women: The Key to Food Security. Food Policy Report.

[97] Doss C. (2018). Women and Agricultural Productivity: Reframing the Issues. Dev. Policy Rev..

[98] FAO, IFAD, UNICEF, WFP, WHO (2023). The State of Food Security and Nutrition in the World 2023: Urbanization, Agrifood Systems Transformation and Healthy Diets Across the Rural–Urban Continuum.

[99] Global Panel on Agriculture and Food Systems for Nutrition (2020). Future Food Systems: For People, Our Planet, and Prosperity.

[100] Gillespie S., Poole N., van den Bold M., Bhavani R.V., Dangour A.D., Shetty P. (2019). Leveraging agriculture for nutrition in South Asia: What do we know, and what have we learned?. Food Policy.

[101] Fanzo J., Bellows A.L., Spiker M.L., Thorne-Lyman A.L., Bloem M.W. (2021). The Importance of Food Systems and the Environment for Nutrition. Am. J. Clin. Nutr..

[102] Kennedy G., Ballard T., Dop M.C. (2011). Guidelines for Measuring Household and Individual Dietary Diversity.

[103] Arimond M., Wiesmann D., Becquey E., Carriquiry A., Daniels M.C., Deitchler M., Fanou-Fogny N., Joseph M.L., Kennedy G., Martin-Prevel Y. (2010). Simple Food Group Diversity Indicators Predict Micronutrient Adequacy of Women’s Diets in Five Diverse, Resource-Poor Settings. J. Nutr..

[104] Béné C., Oosterveer P., Lamotte L., Brouwer I.D., de Haan S., Prager S.D., Talsma E.F., Khoury C.K. (2019). When Food Systems Meet Sustainability—Current Narratives and Implications for Actions. World Dev..

[105] Allen T., Prosperi P., Cogill B., Flichman G. (2014). Agricultural Biodiversity, Social-Ecological Systems and Sustainable Diets. Proc. Nutr. Soc..

[106] Bruening M., Dinour L.M., Chavez J.B.R. (2017). Food Insecurity and Emotional Health in the USA: A Systematic Narrative Review of Longitudinal Research. Public Health Nutr..

[107] Berkowitz S.A., Basu S., Gundersen C., Seligman H.K. (2019). State-Level and County-Level Estimates of Health Care Costs Associated with Food Insecurity. Prev. Chronic Dis..

[108] Development Initiatives (2024). 2024 Global Nutrition Report: Nourishing the SDGs.

[109] United Nations Food Systems Coordination Hub (2023). Food Systems Transformation Pathways for Sustainable Development.

[110] Searchinger T., Waite R., Hanson C., Ranganathan J., Dumas P., Matthews E. (2019). Creating a Sustainable Food Future: A Menu of Solutions to Feed Nearly 10 Billion People by 2050.

[111] United Nations Environment Programme (UNEP) (2024). Food Waste Index Report 2024.

[112] Hawkes C., Ruel M.T., Salm L., Sinclair B., Branca F. (2020). Double-Duty Actions: Seizing Programme and Policy Opportunities to Address Malnutrition in All Its Forms. Lancet.

[113] FAO, WHO (2019). Healthy Diets from Sustainable Food Systems: Food-Based Dietary Guidelines and Food Policies.

[114] Abdoul-Azize H.T., El Gamil R. (2021). Social protection as a key tool in crisis management: Learnt lessons from the COVID-19 pandemic. Glob. Soc. Welf..

[115] United Nations (2015). Transforming Our World: The 2030 Agenda for Sustainable Development.

